# Transboundary risk of African swine fever (ASF): Detection of ASF virus genotype II in pork products carried by international travelers to Indonesia

**DOI:** 10.14202/vetworld.2025.280-286

**Published:** 2025-02-13

**Authors:** Atik Ratnawati, Risza Hartawan, Indrawati Sendow, Muharam Saepulloh, Sumarningsih Sumarningsih, Dyah Ayu Hewajuli, Nuryani Zainuddin, Ni Luh Putu Indi Dharmayanti, I. Wayan Teguh Wibawan, Ni Luh Putu Ika Mayasari

**Affiliations:** 1 Division of Medical Microbiology, School of Veterinary Medicine and Biomedical Sciences, Bogor Agricultural University (IPB University), Bogor, 16680, Indonesia; 2 Research Center for Veterinary Science, Research Organization for Health, National Research and Innovation Agency, Cibinong, 16911, Indonesia; 3 Directorate General Livestock and Animal Health Services, Ministry of Agriculture, Jakarta, 12011, Indonesia

**Keywords:** African swine fever, ASFV, Indonesia, international airports, molecular identification, pork product contamination

## Abstract

**Background and Aim:**

African swine fever (ASF), a devastating viral disease in swine caused by ASF virus (ASFV), has led to substantial economic losses, particularly in Asia since 2018. ASFV’s resilience in diverse environments renders the movement of infected pork products a critical risk for disease transmission. This study aimed to identify ASFV contamination in pork products brought by international travelers to Indonesia, highlighting potential pathways for ASF introduction.

**Materials and Methods:**

From 2019 to 2020, pork food products confiscated at three Indonesia international airports (Soekarno-Hatta, Raja Haji Fisabilillah, and Sultan Aji Muhammad Sulaiman Sepinggan) underwent testing. ASFV detection employed TaqMan real-time polymerase chain reaction targeting the *B646L* (p72) gene, followed by gene sequencing of *B646L* (p72) and *E183L* (p54) for molecular characterization. Phylogenetic analyses were conducted to compare local ASFV strains with global counterparts.

**Results:**

Among 29 confiscated samples, two pork products originating from China tested positive for ASFV. These were identified as genotype II, consistent with strains from Africa, Europe, and Asia. Sequence analyses confirmed the Indonesian strain’ close genetic relationship with global ASFV genotype II isolates, such as those from China, Vietnam, and Georgia.

**Conclusion:**

The presence of ASFV in imported pork products emphasizes the risk posed by international travelers in introducing the virus to ASF-free regions. This underscores the need for stringent border biosecurity measures, surveillance, and public awareness to prevent ASFV outbreaks in Indonesia. Although ASFV does not pose a direct threat to human health, its transmission through swill-feeding practices remains a critical concern for the pig industry.

## INTRODUCTION

African swine fever (ASF) is a severe hemorrhagic disease in both domestic pigs (*Sus scrofa domesticus*) and wild boar (*Sus scrofa ferus*). It is caused by ASF virus (ASFV), a linear double-stranded DNA belonging to the *Asfarviridae* family [1, 2]. The disease presentations of infected pigs include severe hyperthermia, hind leg weakness, recumbence, dyspnea, respiratory distress, diarrhea, anorexia, and vomiting, as well as skin erythema and cyanosis [3, 4]. Subsequently, postmortem examinations revealed that the internal cavities were filled with blood-tinged discharge, including in the *pleura*, *pericardium*, and peritoneum. Signs of inflammation, such as swelling, bleeding, and *petechiae*, are observed in multiple internal organs, including the heart, kidney, liver, intestines, and lymph nodes. The morbidity and mortality rate of acute or peracute onset are very high. ASF is exacerbated by the absence of effective vaccines to control the disease [5, 6]. Therefore, ASF outbreaks not only impact pig farms and industries in terms of food security but also cause significant concerns regarding wildlife conservation because wild boars are an essential part of the food chain in the wildlife ecosystem [7–9].

After its initial reports in Kenya in 1921, ASFV became endemic in many African countries [2]. Later, between 1950 and 1980, this transboundary disease spreads widely to Europe and the Americas. Persistent infection in the field has caused virus evolution, resulting in substantial genetic variants. In 2007, ASFV was reported in Georgia, outside Africa, where it rapidly spread to neighboring countries. ASFV genotype II was first recorded in China in August 2018 and was subsequently reported in other Asian countries [10, 11]. By 2018, approximately 24 genotypes of ASFV had been identified worldwide, specifically in Africa [10]. A year later, ASF outbreaks were reported in neighboring countries, such as Mongolia, Vietnam, Cambodia, North Korea, Laos, Myanmar, Timor-Leste, the Philippines, South Korea, and Indonesia. In Indonesia, numerous ASF outbreaks have been reported in several regions, including Sumatra, Java, Bali, and Nusa Tenggara islands [12–14]. Based on molecular analysis, ASFV in Indonesia is genotype II and is similar to ASFV strains from other countries, such as Vietnam, China, and Russia [13].

The disease can be transmitted either through direct transmission through infected animals and body secretion/excretion or through indirect transmission through contaminated fomites or pork products. Ticks of *Ornithodoros moubata* also transmit ASFV [11, 15, 16]. ASFV is relatively stable in the environment and can be maintained for an extended period in animal products and carcasses [17]. ASFVs can also survive for up to 83 days in various dry-cured pork meat products, although still within the shelf-life of the products [18]. In Taiwan and South Korea, pork products originating from China have been shown to be positive for ASFV [19, 20]. Therefore, based on this evidence, a plausible route for the introduction of ASF into new areas/regions/countries is through contaminated pork products brought by people coming from ASFV-infected areas.

This study aimed to investigate ASFV contamination in pork products originating from outside of Indonesia at international airports during ASF incursion (initial introduction) in Indonesia, following the guidelines of the Indonesian Center Quarantine Agency. Because ASFV was identified in contaminated pork products brought by travelers, this study identified a potential source of ASF introduction into Indonesia, but this has not been confirmed.

## MATERIALS AND METHODS

### Ethical approval

This research was approved by the Animal Welfare Commission of the Indonesian Agricultural Research and Development Agency (approval number: Balitbangtan/BB Litvet/01/NRm/2020.

### Study period and location

The study was conducted using contaminated pork products collected from September 2019 to January 2020 at three Indonesian international airports: The Soekarno-Hatta International Airport (Cengkareng, West Jakarta, DKI Jakarta Province), the Raja Haji Fisabilillah Airport (Tanjung Pinang, Riau Province), and the Sultan Aji Muhammad Sulaiman Sepinggan Airport (Balikpapan, East Kalimantan Province). Laboratory investigations were undertaken at the Virology Department of the Research Center for Veterinary Science under the Ministry of Agriculture, Bogor, Indonesia. The Joint Food and Agriculture Organization of the United Nations/International Atomic Energy Agency (FAO/IAEA) Agriculture and Biotechnology Laboratory, Austria, as the reference laboratory, were conducted sequencing for the polymerase chain reaction (PCR) positive samples.

### Sample collection and processing

Various types of pork products were confiscated from incoming travelers at the Soekarno–Hatta International Airport, the Raja Haji Fisabilillah Airport, and the Sultan Aji Muhammad Sulaiman Sepinggan Airport by the Indonesian Center of Quarantine Agency for passive surveillance. We transported the samples to the Virology Laboratory of the Research Center for Veterinary Science in Bogor, West Java, in sealed containers on ice packs. In the laboratory, approximately 5 g of sample was homogenized in 2 mL of sterile phosphate saline buffer. The homogenate was then centrifuged at 1000× *g* for 10 min, and the supernatant was stored at −20°C for further analyses.

### ASFV detection

ASFV was identified using real-time PCR (qPCR) as designed by King *et al*. [21] with few modifications on the reagents and concentration of primers and probes. The primers and probes used were 5’ CTGCTCATGGTATCAATCTTATCGA 3’, 5’ GATACCACAAGATCRGCCGT 3’, and FAM-GATACCACAAGATCRGCCGT-BHQ1. The viral DNA was isolated from the supernatant of the homogenized samples (see above) using a DNeasy Mini Kit (Qiagen, Germany) according to the manufacturer’s instructions. DNA ASFV for positive control was provided by the IAEA and No Template Control (NTC) contained nuclease-free water for negative control. qPCR was performed using iTaq Universal Probes Supermix (Bio-Rad, USA, catalog no. 1725130). Briefly, 2 µL of the template DNA was added to a final volume of 20 µL consisting of 1 × iQ Supermix, 400 nM of each primer, and 250 nM of probe. qPCR was performed using aCFX96 Touch Real-Time PCR Detection System (Bio-Rad). The thermal profile consisted of initial denaturation at 95°C for 5 min and 40 amplification cycles (95°C for 15 s and 58°C for 30 s). Fluorescence was read at 58°C. The amplification was considered positive at a Cq value of ≤35 and negative at a Cq value of ≥40.

### Molecular characterization of ASFV

The qPCR-positive samples were sent to the FAO/IAEA Animal Production and Health Laboratories (APHL), Austria, for confirmation. The molecular characterization was conducted by sequencing the ASF viral genes, namely the *B646L* gene (p72) and *E183L* gene (p54), using previously described methodologies by Bastos *et al*. [22], Gallardo *et al*. [23], and Mulumba-Mfumu *et al*. [24]. Subsequently, phylogenetic tree analysis was performed to analyze the molecular characteristics of these gene sequences based on previous studies by Couacy-Hymann *et al*. [3] and Dharmayanti *et al*. [13] using MEGA 6 software (https://www.megasoftware.net/).

## RESULTS

**Figure 1:**
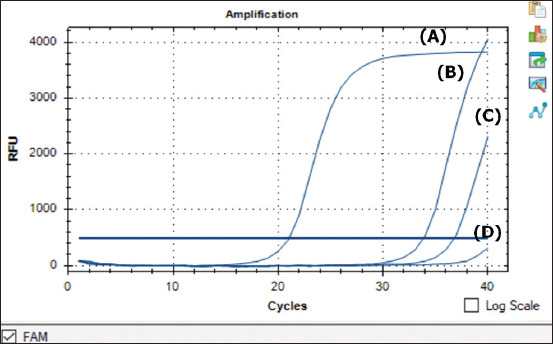
Identification of contaminated African swine fever virus in pork product samples using real-time polymerase chain reaction. The Cq values were 21.03, 33.82, 36.74, and none. (A) for the positive control, (B) Indonesia/2019/T04, (C) Indonesia/2019/T05, and (D) negative control, respectively.

Twenty-nine pork product samples from three Indonesian international airports were tested for ASFV using qPCR based on the *B646L* (p72) gene. Detailed information about the samples, including the type of pork, country of origin, and entry point, is provided in Table 1. Two samples originating from China in September 2019 at the Soekarno–Hatta International Airport (Cengkareng, West Jakarta, DKI Jakarta Province) were positive for ASFV, namely sample 007 (raw meat) and sample 017 (smoked ham), with Cq values of 33.82 and 36.74, respectively (Table 1 and Figure 1). These samples were named Indonesia/2019/T04 and Indonesia/2019/T05 for further identification. On the other hand, the samples that originated from South Korea and arrived at the same international airport were negative for ASFV. At the same time, the samples that originated from Singapore and arrived at the Raja Haji Fisabilillah Airport (Tanjung Pinang, Riau Province) and the Sultan Aji Muhammad Sulaiman Sepinggan Airport (Balikpapan, East Kalimantan Province) in September 2019 and January 2020 were also negative for ASFV.

**Table 1:** Detection of ASFV in pork products at three Indonesian international airports during 2019 and 2020.

Sample code	Month and year of the sampling	Pork products	Country origin	Airport	Real-time PCR
001	September 2019	Raw meat	China	West Jakarta^1^	Negative
002	September 2019	Sausage	China	West Jakarta^1^	Negative
003	September 2019	Sausage	South Korea	West Jakarta^1^	Negative
004	September 2019	Ham	South Korea	West Jakarta^1^	Negative
005	September 2019	Sausage	China	West Jakarta^1^	Negative
006	September 2019	Raw meat	China	West Jakarta^1^	Negative
007	September 2019	Raw meat	China	West Jakarta^1^	Positive
008	September 2019	Sausage	China	West Jakarta^1^	Negative
009	September 2019	Sausage	China	West Jakarta^1^	Negative
010	September 2019	Sausage	China	West Jakarta^1^	Negative
011	September 2019	Raw meat	China	West Jakarta^1^	Negative
012	September 2019	Raw meat	China	West Jakarta^1^	Negative
013	September 2019	Raw meat	China	West Jakarta^1^	Negative
014	September 2019	Sausage	China	West Jakarta^1^	Negative
015	September 2019	Sausage	China	West Jakarta^1^	Negative
016	September 2019	Intestine	China	West Jakarta^1^	Negative
017	September 2019	Smoked ham	China	West Jakarta^1^	Positive
018	September 2019	Smoked ham	China	West Jakarta^1^	Negative
019	September 2019	Sausage	China	West Jakarta^1^	Negative
020	December 2019	Raw meat	Singapore	Balikpapan^2^	Negative
021	January 2020	Raw meat	Singapore	Tanjung Pinang^3^	Negative
022	January 2020	Raw meat	Singapore	Tanjung Pinang^3^	Negative
023	January 2020	Raw meat	Singapore	Tanjung Pinang^3^	Negative
024	January 2020	Raw meat	Singapore	Tanjung Pinang^3^	Negative
025	January 2020	Sausage	Singapore	Tanjung Pinang^3^	Negative
026	January 2020	Sausage	Singapore	Tanjung Pinang^3^	Negative
027	January 2020	Sausage	Singapore	Tanjung Pinang^3^	Negative
028	January 2020	Sausage	Singapore	Tanjung Pinang^3^	Negative
029	January 2020	Raw meat	Singapore	Tanjung Pinang^3^	Negative

1 The Soekarno–Hatta International Airport, Cengkareng, West Jakarta, DKI Jakarta Province,

2 Sultan Aji Muhammad Sulaiman Sepinggan Airport, Balikpapan, Indonesia,

3 Raja Haji Fisabilillah Airport, Tanjung Pinang, Riau Province, Thailand. ASFV=African swine fever virus, PCR=Polymerase chain reaction

Subsequently, APHL conducted in Austria confirmed the positive results of ASFV identification. Furthermore, molecular identification by gene sequencing of *B646L* (p72) and *E183L* (p54) from the positive samples confirmed the evidence of a previous diagnosis by qPCR for ASFV. The gene sequences of the Indonesian ASFV strain from pork products were submitted to the NCBI GenBank database under accession numbers, *i.e*., *B646L* (p72) (GenBank accession nos. PP869832 and PP869833) and *E183L* (p54) (GenBank accession nos. PP869834 and PP869835).

**Figure 2:**
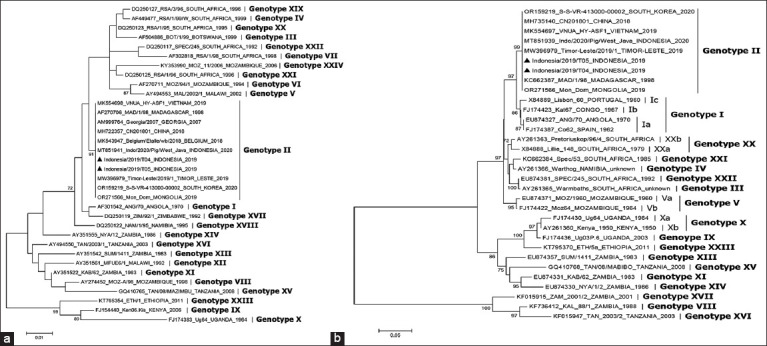
The phylogenetic trees illustrate the relationship between the African swine fever virus strains within the study (black triangle marking) in the virus genotype constellation. The trees based on the *B646L* (p72) gene (a) and *E183L* (p54) gene (b) were constructed using the maximum likelihood and minimum evolution methods, respectively. Only bootstrap values >70% are shown.

Regardless of the country of origin, Basic Local Alignment Search Tool Nucleotide (BLASTN) and phylogenetic tree analysis demonstrated that the *B646L* (p72) and *E183L* (p54) gene sequences of the Indonesian ASFV were identical to genotype II ASFV strains from other countries in Africa, Europe, and Asia, such as Georgia, China, Mongolia, South Korea, Vietnam, Thailand, Malaysia, and Timor-Leste (Figure 2). The phylogenetic tree analyses based on the *B646L* (p72) and *E183L* (p54) genes demonstrated that the ASFV strains isolated in this study clustered with other ASFV strains belonging to genotype II (Figure 2).

## DISCUSSION

Since its initial emergence in August 2018, ASF outbreaks caused by genotype II variants have spread from China to neighboring countries, causing the deaths of thousands of pigs and threatening global food security [11, 25]. The rapid and massive spread of human-driven viral diseases throughout many countries involves numerous factors, including intensive industrial systems and the movement and trading of animals and their products [26]. In ASF cases, indirect disease transmission is common because ASFV appears to be relatively stable in the environment, including in food products originating from pigs [27]. Therefore, travelers who carry pork products from infected areas may become a significant risk introduction to naïve areas. The coronavirus-19 outbreaks demonstrated that in this globalization era, international airline pathways may also play a crucial role in the rapid spread of ASF disease in multiple regions/countries rapidly [28, 29].

This study confirmed ASFV contamination of pork products carried by Chinese airline passengers in September 2019 at Jakarta International Airport. Other studies in Taiwan and South Korea have reported similar outcomes for ASFV contamination of pork products brought by travelers from China [19, 20]. Furthermore, previous studies by Petrini *et al*. [18], Mebus *et al*. [30], and Mebus *et al*. [31] have confirmed the long-term survival of ASFV in dry-cured pork meats such as ham, sausage, and salami. For unprocessed food such as raw meat, the viability of infective ASFV should be higher than that of pork dry-cured meat since no treatments can cause virus inactivation. Surprisingly, ASFV may also survive in trading items for animal husbandry, such as complete feed and feed additives, even in feed ingredients based on plants [32, 33].

ASFV is not a zoonotic threat; therefore, pork products contaminated with ASFV do not pose a threat to human health [11]. However, contaminated pork products constitute a significant risk factor for indirect disease transmission due to swill feeding practices with contaminated pork products, especially in small pig-holder farms. This practice is common in many countries including Indonesia, because of socioeconomic reasons [34]. Consequently, several ASF outbreaks in African and Asian countries were predicted to occur due to swill-feeding practices [35–37]. Therefore, it is possible that contaminated pork products carried by travelers from infected countries can be used as swill-feeding in pig farms in destined countries. These pork products pose a low potential for short-term risks when fed to pigs. Without appropriate treatment, contaminated pork products can become a primary source of infection in disease-free countries [34].

Because no effective vaccine is available, a practical approach to controlling ASF is to implement biosecurity measures to manage porcine husbandry. Moreover, biosecurity practices should also be implemented for other diseases, such as foot and mouth diseases, influenza, classical swine fever, and others [38, 39]. However, essential mitigation to protect national pig industries is preventing the entry of exotic diseases since recent outbreaks of ASF by genotype I have been reported in China [40, 41]. Therefore, imported items related to pigs and their products from ASF-reported countries should be ensured to be free from viruses by implementing appropriate methods, such as strict border security practices and ASFV identification tests based on molecular approaches.

## CONCLUSION

This study successfully identified ASFV contamination in pork products carried by international travelers to Indonesia, underscoring a potential route for disease introduction. Using TaqMan-based real-time PCR and confirmatory sequencing of *B646L* (p72) and *E183L* (p54) genes, ASFV genotype II, consistent with strains circulating in Asia, Europe, and Africa, was detected in two samples originating from China. The genetic similarity to global genotype II strains highlights the transboundary nature of ASFV and its threat to the swine industry and food security. The study demonstrates significant strengths, including its methodological rigor through the use of molecular diagnostics, ensuring high specificity and sensitivity in ASFV detection. The global relevance of identifying a direct pathway for ASFV introduction through contaminated pork products provides actionable insights into mitigating disease transmission risks in a globalized context. In addition, the comprehensive sampling framework across three major international airports adds depth to the findings. However, there are limitations. The relatively small sample size of 29 may not fully represent the diversity of pork products or the extent of ASFV contamination in travelers’ luggage. The restricted geographic scope of focusing solely on Indonesian airports limits the broader applicability of the finding. Furthermore, the study lacks a detailed epidemiological context, such as analysis of risk factors like swill feeding practices, which could provide a more comprehensive understanding of ASFV transmission dynamics. Future studies could benefit from larger-scale surveillance with increased sample size and broader geographic coverage to improve generalizability. Investigating socioeconomic factors, such as feeding practices, would offer valuable insights into the pathways through which contaminated pork products contribute to ASF outbreaks. The development of real-time molecular diagnostic systems at border checkpoints would enhance early detection and response strategies. Furthermore, these findings can inform stricter border biosecurity policies, traveler education programs, and enhanced detection protocols to prevent the introduction of exotic animal diseases. By addressing its limitations and building on its strengths, this study provides a solid foundation for further research and practical interventions to safeguard the global swine industry and ensure food security.

## AUTHORS’ CONTRIBUTIONS

AR, NZ, and IS: Supervised the field sampling, data collection, and laboratory work. AR, IS, RH, MS, SS, DAH, and NLPIM: Data entry, analysis, and interpretation and drafted the manuscript. IS, AR, NZ, RH, NLPIM, NLPID, and IWTW: Conceptualized and designed the study and reviewed and edited the manuscript. All authors have read and approved the final version of the manuscript.
